# Application of artificial intelligence in the health management of chronic disease: bibliometric analysis

**DOI:** 10.3389/fmed.2024.1506641

**Published:** 2025-01-07

**Authors:** Mingxia Pan, Rong Li, Junfan Wei, Huan Peng, Ziping Hu, Yuanfang Xiong, Na Li, Yuqin Guo, Weisheng Gu, Hanjiao Liu

**Affiliations:** ^1^School of Nursing, Fujian University of Traditional Chinese Medicine, Fuzhou, China; ^2^Shenzhen Hospital of Integrated Traditional Chinese and Western Medicine, Shenzhen, China; ^3^Department of Neurology, People’s Hospital of Longhua, Shenzhen, China; ^4^Seventh Clinical Medical College, Guangzhou University of Chinese Medicine, Shenzhen, China; ^5^School of Nursing, Guangzhou University of Chinese Medicine, Guangzhou, China

**Keywords:** artificial intelligence, chronic disease, health management, nursing care, bibliometric analysis

## Abstract

**Background:**

With the rising global burden of chronic diseases, traditional health management models are encountering significant challenges. The integration of artificial intelligence (AI) into chronic disease management has enhanced patient care efficiency, optimized treatment strategies, and reduced healthcare costs, providing innovative solutions in this field. However, current research remains fragmented and lacks systematic, comprehensive analysis.

**Objective:**

This study conducts a bibliometric analysis of AI applications in chronic disease health management, aiming to identify research trends, highlight key areas, and provide valuable insights into the current state of the field. Hoping our findings will serve as a useful reference for guiding further research and fostering the effective application of AI in healthcare.

**Methods:**

The Web of Science Core Collection database was utilized as the source. All relevant publications from inception to August 2024 were retrieved. The external characteristics of the publications were summarized using HistCite. Keyword co-occurrences among countries, authors, and institutions were analyzed with Vosviewer, while CiteSpace was employed to assess keyword frequencies and trends.

**Results:**

A total of 341 publications were retrieved, originating from 775 institutions across 55 countries, and published in 175 journals by 2,128 authors. A notable surge in publications occurred between 2013 and 2024, accounting for 95.31% (325/341) of the total output. The United States and the Journal of Medical Internet Research were the leading contributors in this field. Our analysis of the 341 publications revealed four primary research clusters: diagnosis, care, telemedicine, and technology. Recent trends indicate that mobile health technologies and machine learning have emerged as key focal points in the application of artificial intelligence in the field of chronic disease management.

**Conclusion:**

Despite significant advancements in the application of AI in chronic disease management, several critical challenges persist. These include improving research quality, fostering greater international and inter-institutional collaboration, standardizing data-sharing practices, and addressing ethical and legal concerns. Future research should prioritize strengthening global partnerships to facilitate cross-disciplinary and cross-regional knowledge exchange, optimizing AI technologies for more precise and effective chronic disease management, and ensuring their seamless integration into clinical practice.

## Introduction

Chronic diseases are defined as disorders that persist for a long period, typically lasting more than 3 months, and are generally considered incurable. These diseases are characterized by high prevalence, morbidity, mortality, and disability (1). According to the World Health Organization, chronic diseases account for 41 million deaths annually, nearly 74% of all global deaths, with cardiovascular diseases, cancer, chronic respiratory diseases, and diabetes responsible for approximately 33.3 million deaths (2). Furthermore, the socioeconomic burden of chronic diseases is substantial, encompassing both direct medical costs and indirect costs, such as loss of productivity, caregiving expenses, and economic losses due to premature death. A study in the United States found that direct medical costs associated with chronic diseases account for 86% of total healthcare expenditures (3). As the incidence of chronic diseases continues to rise, their economic impact has become increasingly significant, particularly for low-and middle-income countries and regions, further exacerbating their economic burden. In summary, chronic diseases not only pose a threat to individual health but also have widespread adverse effects, including medical burdens and economic pressures globally. Therefore, comprehensive and systemic measures are urgently required to alleviate the escalating burden they impose.

Health management refers to the process of helping patients effectively improve their health through comprehensive monitoring, assessment, intervention, and maintenance of their health status (4). Specifically, its core objectives are to alleviate the occurrence and development of diseases, reduce healthcare costs, and improve the overall quality of life. In practice, health management not only targets individuals but also groups, aiming to enhance self-management awareness and abilities through health education. Moreover, it utilizes health information collection, testing, assessment, and personalized management programs, along with interventions for lifestyle-related health risk factors, to continuously improve health outcomes (5). Research has consistently shown that health management can significantly reduce the incidence, morbidity, and mortality of chronic diseases by employing early screening, risk assessment, personalized interventions, and long-term follow-up (6). As a result, to address the growing public health crisis caused by chronic diseases, many countries are actively building comprehensive health management systems (7). Nevertheless, due to the ongoing shortage of medical resources, the health management needs of patients with chronic diseases have yet to be fully met.

Artificial intelligence (AI) is a field of study that simulates and extends human cognitive functions through computer technology, with the goal of assisting or augmenting human capabilities in complex tasks (8). Since its introduction in 1956, the application of AI in healthcare has continuously expanded, encompassing areas such as patient care, medical education, and clinical decision-making (9, 10). More specifically, in chronic disease management, AI enables comprehensive management, from early screening to ongoing care, thereby enhancing the precision and efficiency of disease prevention and treatment (11). While research demonstrates that AI can enhance patients’ quality of life and reduce strain on healthcare resources by optimizing health management strategies and improving chronic disease risk diagnosis (12–15), there is still considerable uncertainty regarding AI’s full role in chronic disease management (16–18). Although some progress has been made in integrating AI into this field, the research topics, patterns, and trends related to AI-driven chronic disease management remain poorly defined. This lack of clarity presents challenges for researchers, policymakers, and healthcare providers seeking to identify the most effective AI applications in chronic disease management. One significant gap in the current body of research is the absence of comprehensive bibliometric analyses specifically focused on this intersection of AI and chronic disease management (19).

Bibliometrics, as a research tool, provides valuable insights through the application of mathematical and statistical methods to analyze published literature. It helps to map out research trends, publication patterns, and the relationships between various studies in a given field (20, 21). By identifying influential research works, emerging themes, and collaboration networks, bibliometric analysis can clarify the development of a particular research domain. Given the unclear research direction and limited systematic evaluations in AI-driven chronic disease management, conducting a bibliometric analysis is critical. This analysis will help identify gaps, guide future research efforts, and contribute to a more comprehensive understanding of how AI can be applied effectively to improve chronic disease outcomes. Hence, this study aims to fill the void by performing a bibliometric analysis in this area, providing an essential foundation for subsequent research. This study employs bibliometric methods to analyze the current landscape of research on the application of AI in chronic disease health management. By doing so, it seeks to identify prevailing research trends, key topics, and emerging areas of interest. Instead of attempting to cover every aspect of AI’s impact on chronic disease management, this study focuses on providing a comprehensive overview of current research trends, identifying key gaps, and suggesting potential directions for future exploration. Through this refined approach, the study aims to offer actionable insights for researchers, guiding them toward promising areas of further exploration. Additionally, the findings provide practical implications for policymakers, healthcare providers, and technology companies, helping to inform decision-making processes and optimize the integration of AI in healthcare systems.

## Materials and methods

### Data sources and search strategy

Relevant literature was retrieved from the Web of Science Core Collection (WoSCC), which is a comprehensive and influential global scientific database (22). The search covered the period from the database’s inception to August 2024. The search terms are as follows: (“artificial intelligence” OR “AI” OR “machine intelligence” OR “machine learning” OR “deep learning” OR “data learning” OR “data mining” OR “big data” OR “intelligent systems” OR “intelligent learning” OR “feature* learning” OR “supervised learning” OR “supervised machine learning” OR “robot*” OR “robot technology” OR “assistant robot” OR “robot-assisted” OR “computational intelligence” OR “computer reasoning” OR “computer vision system” OR “sentiment analysis” OR “decision tree*” OR “mHealth” OR “e-health” OR “mobile health” OR “telehealth” OR “telemedicine” OR “digital health tools” OR “digital monitoring” OR “health information technology”) AND (“chronic disease” OR “chronic illness” OR “chronic condition” OR “long-term illness” OR “long-lasting disease” OR “Prolonged disorder”) AND (“health management” OR “health administration” OR “health maintenance” OR “health supervision” OR “healthcare management” OR “disease management” OR “community health management”).

Inclusion criteria:

1. Research articles focusing on the application of AI technologies in chronic disease management, with no restrictions on the type of study.
2. For duplicate publications, the earliest published and most complete version was included.
3. Only articles published in English were included.

Exclusion criteria:

1. News articles, conference papers, reviews, and retracted publications.
2. Articles with only abstracts and no access to full text.
3. Case reports and books.
4. Publications with incomplete information or those unrelated to the research topic.

After the initial search, two researchers independently screened and assessed all relevant literature to ensure its pertinence to the study topic. In case of disagreement, a third investigator was consulted for resolution.

### Data analysis and network mapping

In this study, a comprehensive bibliometric analysis was conducted using HistCite, VOSviewer, and CiteSpace, each serving specific analytical purposes. Firstly, HistCite, developed by Eugene Garfield, the inventor of the Science Citation Index (SCI), was used to analyze the historical evolution of citations within the field. HistCite is a robust citation analysis tool that allows the identification of the most influential authors, journals, and papers (23). In this research, HistCite Pro 2.1 was used to generate relevant tables that systematically documented the progression of the literature, providing a clear overview of how the field has evolved over time. VOSviewer, a Java-based software developed by the Centre for Science and Technology Studies at Leiden University, was utilized for mapping and visualizing scientific knowledge. This software excels at handling large-scale bibliometric data and generating various network analyses, including co-occurrence networks, citation networks, and term frequency analyses (24). In this study, VOSviewer 1.6.20 was used to analyze collaboration networks and visualize keyword co-occurrence, helping identify core themes, trends, and key publications in the field. Lastly, CiteSpace, developed by Chaomei Chen, was employed for a deeper analysis of scientific literature. This open-source software specializes in constructing science maps that reveal research hotspots, knowledge foundations, and emerging trends (25). In this research, CiteSpace 6.3.R1 was employed to detect keyword bursts, identifying evolving research trends.

### Ethical considerations

The data utilized in this study were sourced from the WoSCC, and no involvement from patients or public contributors was included in this research.

## Results

### Analysis of publication outputs and the total local citation score

**Figure 1:**
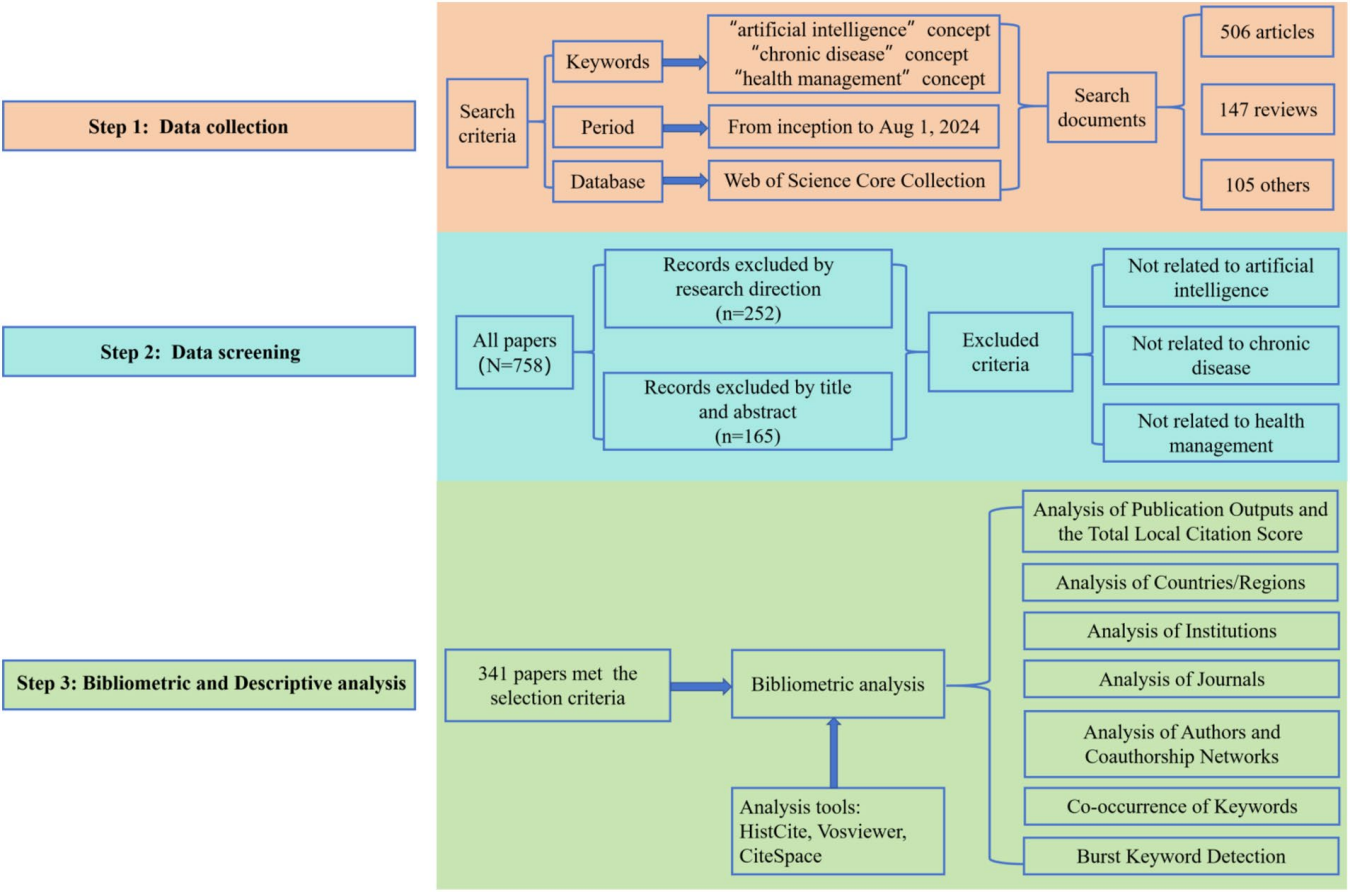
Flowchart of the literature-screening process and research framework.

**Figure 2:**
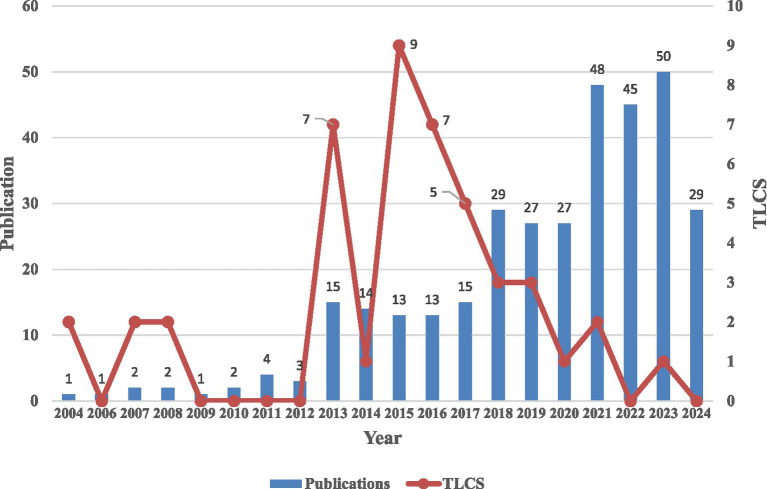
Publication output and the TLCS over time. TLCS, total local citation score.

A total of 758 articles were retrieved from the WoSCC, and after data cleaning, 341 articles (4.50%) were included for analysis. Figure 1 presents a comprehensive overview of the literature screening process and the framework for visualized analysis. Additionally, Figure 2 depicts the annual publication trends. Initially, from 2004 to 2012, the average annual number of publications remained relatively low, with fewer than five articles published each year. However, starting in 2013, there was a marked increase in publications, with over 10 articles published annually from 2013 to 2017. Furthermore, between 2018 and 2024, the number of publications surged rapidly, peaking at 50 articles in 2023. It is important to note that the slight decline in 2024 may be attributed to the search cutoff in August 2024, likely resulting in some articles not yet being indexed at the time of retrieval. Despite the low publication from 2004 to 2012, there were a few citations in 2004, 2007, and 2008, with a Total Local Citation Score (TLCS) of 2. On the other hand, from 2013 to 2017, the TLCS remained high. However, it is worth noting that despite the significant increase in publication volume between 2020 and 2024, the TLCS noticeably declined.

### Analysis of countries/regions

**Figure 3:**
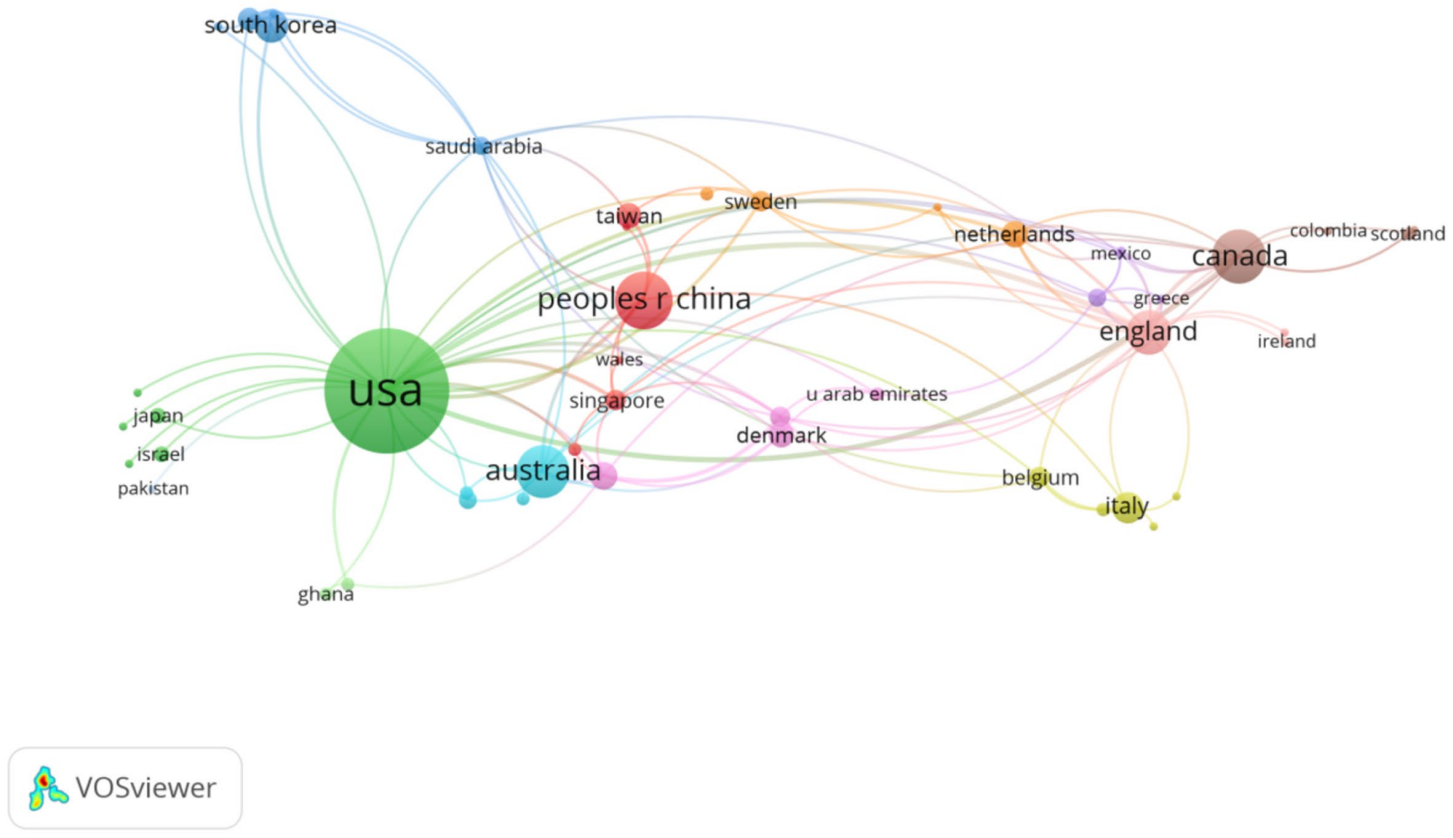
Visualization of research networks of countries/regions.

A total of 55 countries/regions contributed to the publication of the 341 articles. Specifically, Table 1 lists the top 10 countries/regions by publication ranked by the number of publications. Notably, the United States dominated the application of AI in chronic disease health management, with 173 articles published accounting for 50.73% of the total. Meanwhile, China ranked second, with 38 papers (11.14%). In addition, Canada, Australia, and the United Kingdom also contributed a substantial number of publications, with 33, 31 and 24 papers, respectively. Moreover, TLCS and Total Global Citation Score (TGCS) reflect the impact of research in different countries (26). The United States not only led in the number of publications but also had the highest TGCS with 3,634. It is worth noting that although China ranked second in the number of articles published, its TLCS (1 citation) and TGCS (437 citations) were relatively low. The TGCS scores of Canada, Australia, and the U.K. were 542, 592, and 632, respectively. Furthermore, Figure 3 illustrates the collaboration network among countries and regions. As shown in the knowledge map, there was close collaboration between the United States and multiple countries, particularly China, Australia, and Canada.

**Table 1:** Top 10 countries/regions by number of publications.

Rank	Country	Publications, *n* (%)	TLCS^a^	TGCS^b^
1	United States	173 (50.73)	29	3,634
2	China	38 (11.14)	1	437
3	Canada	33 (9.68)	9	542
4	Australia	31 (9.09)	4	592
5	United Kingdom	24 (7.04)	0	632
6	South Korea	13 (3.81)	1	212
7	Italy	11 (3.23)	1	229
8	Germany	9 (2.64)	0	37
9	Netherlands	8 (2.35)	0	173
10	Denmark	7 (2.05)	0	90

^aTLCS, total local citation score.^

^bTGCS, total global citation score.^

### Analysis of institutions

**Figure 4:**
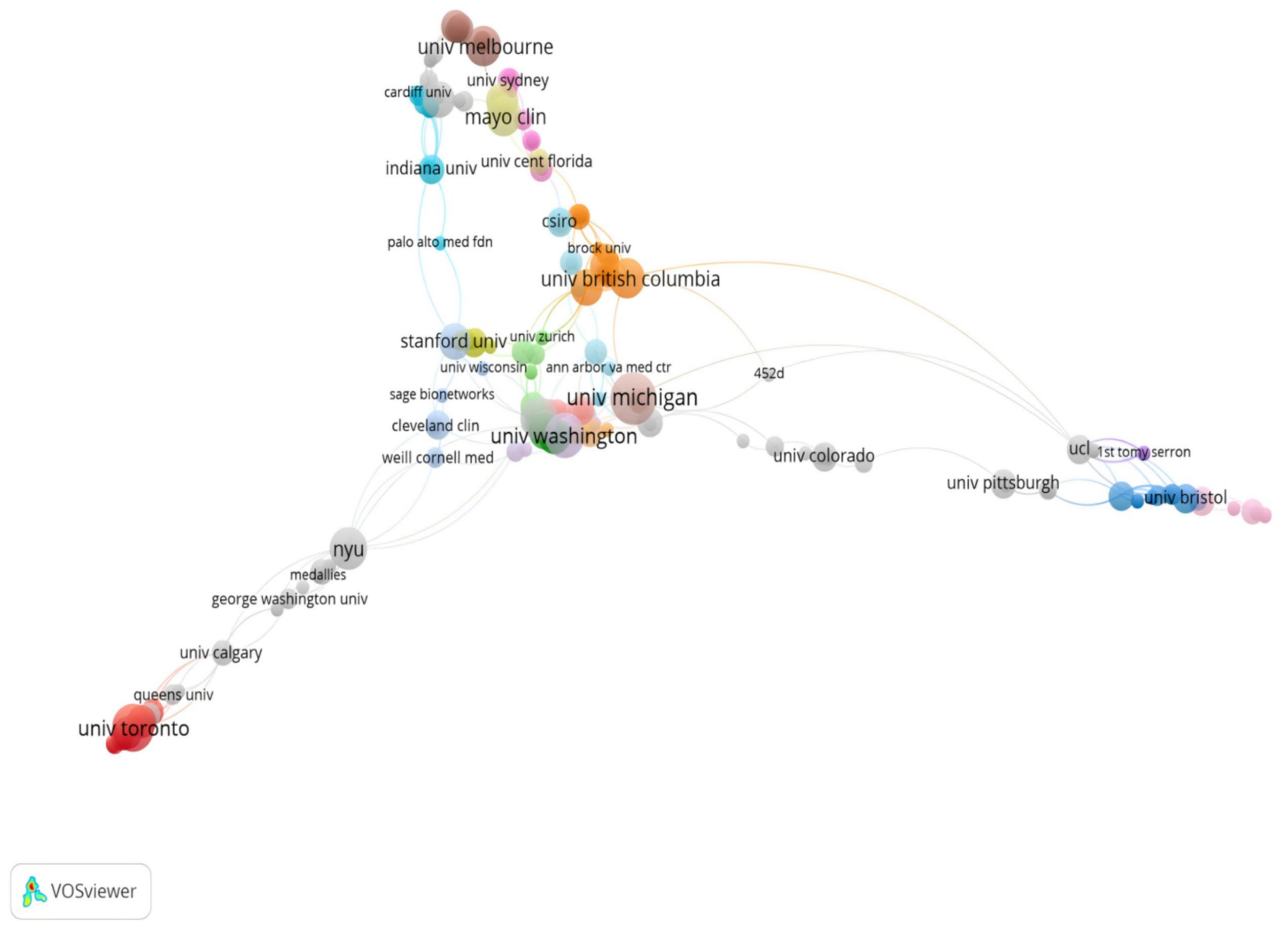
Visualization of research networks of institutions.

A total of 749 institutions contributed to the publication of 341 papers. As shown in Table 2, the top 10 institutions were ranked by the number of publications in this field. Among these top 10 institutions, seven were based in the United States, two in Canada, and one in Australia. Specifically, the University of Michigan ranked first with 12 publications, followed by the University of Toronto with 11, and the University of Washington with 9. Moreover, based on the TLCS and TGCS data provided in the table, the University of Michigan had the highest TGCS of 392. Similarly, the University of Washington had a TGCS of 231. In addition, Figure 4 illustrates the collaborations between institutions. The co-occurrence map clearly shows that the University of Michigan and the University of Washington are at the center of the research network. These two institutions have established strong collaborative ties with multiple other institutions. Furthermore, as shown in the figure, not only are U.S. research institutions central to this network, but Canadian and Australian institutions, such as the University of Toronto and the University of Melbourne, also play prominent roles. Interestingly, despite having a TLCS of 0, the University of Toronto has a high TGCS of 141. Additionally, it is noteworthy that the top 10 institutions include not only universities but also medical research organizations, such as the Mayo Clinic.

**Table 2:** Top 10 institutions by number of publications.

Rank	Institution	Publications, *n* (%)	TLCS^a^	TGCS^b^
1	University of Michigan (United States)	12 (3.52)	2	392
2	University of Toronto (Canada)	11 (3.23)	0	141
3	University of Washington (United States)	9 (2.64)	1	231
4	New York University (United States)	8 (2.35)	1	65
5	University of British Columbia (Canada)	8 (2.35)	4	99
6	University of California, Los Angeles (United States)	8 (2.35)	2	96
7	Mayo Clin (United States)	7 (2.05)	0	40
8	Simon Fraser University (Canada)	7 (2.05)	4	57
9	Stanford University (United States)	7 (2.05)	1	244
10	University of Melbourne (Australia)	7 (2.05)	1	146

^aTLCS, total local citation score.^

^bTGCS, total global citation score.^

### Analysis of journals

Journal analysis plays a crucial role in identifying the core journals within a specific field (27). Table 3 presents a summary of the top 10 journals ranked by publication count. Among these, the *Journal of Medical Internet Research* stood out with the highest number of publications, representing 6.74% (23 papers) of the total. Additionally, this journal has a relatively high impact factor (IF = 5.800), underscoring its prominence in the field. This journal primarily focuses on the intersection of the internet and healthcare, which positions it as a significant influence in the field of artificial intelligence and health management. Furthermore, in terms of TGCS, both the *Journal of Medical Internet Research* and *JMIR mHealth and uHealth* were highly cited, with TGCS values of 674 and 611, respectively, highlighting their global impact, particularly in AI and chronic disease management research. Notably, the impact factors of the *Journal of Medical Internet Research* and *JMIR mHealth and uHealth* (5.800 and 5.400, respectively) are considerably higher compared to other journals in the field. In contrast, when considering TLCS, *Telemedicine and e-Health* and *International Journal of Medical Informatics* scored 8 and 2, respectively.

**Table 3:** Top 10 journals that published papers on the application of AI^a^ in the health management of chronic disease.

Rank	Journal	Publications, *n* (%)	TLCS^b^	TGCS^c^	IF^d^
1	*Journal of Medical Internet Research*	23 (6.74)	0	674	5.800
2	*Telemedicine and e-Health*	17 (4.99)	8	245	2.800
3	*JMIR mHealth and uHealth*	14 (4.11)	0	611	5.400
4	*International Journal of Environmental Research and Public Health*	9 (2.64)	0	82	4.530
5	*International Journal of Medical Informatics*	7 (2.05)	2	164	3.700
6	*JMIR Formative Research*	7 (2.05)	0	9	2.000
7	*Journal of General Internal Medicine*	7 (2.05)	2	150	4.300
8	*PLoS One*	7 (2.05)	0	99	2.900
9	*BMC Health Services Research*	6 (1.76)	0	295	2.700
10	*BMJ Open*	6 (1.76)	0	113	2.400

^aAI, artificial intelligence.^

^bTLCS, total local citation score.^

^cTGCS, total global citation score.^

^d^IF, impact factor (Journal Citation Reports 2023).

### Analysis of authors and coauthorship networks

**Figure 5:**
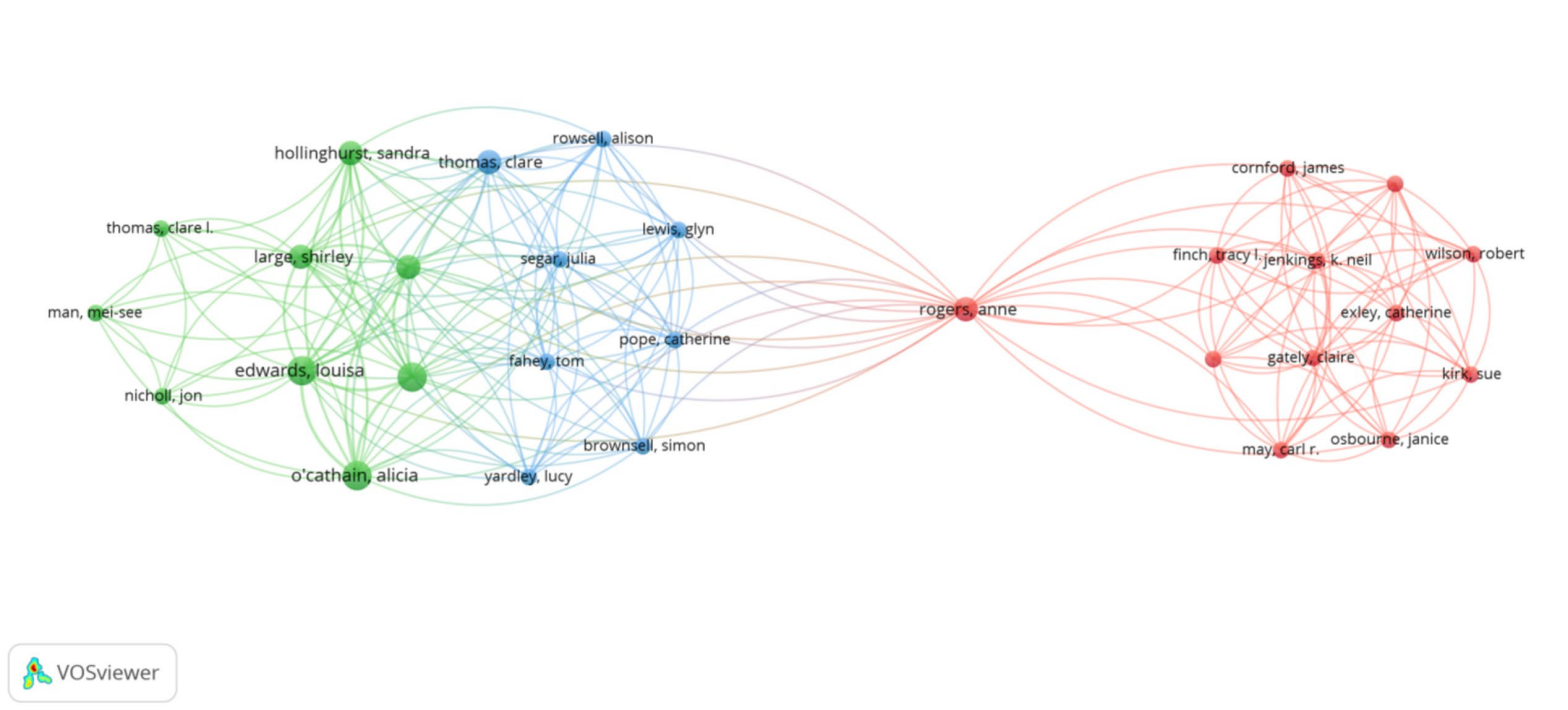
Visualization of research networks of authors.

The high citation index (h-index), a high citation metric, was introduced in 2005 by Jorge E. Hirsch from the University of California, San Diego, as a comprehensive quantitative measure to assess researchers’ academic achievements (28). Table 4 presents the top 10 authors ranked by the number of publications. Notably, several authors from China, including Deng N, Chen J, Li J, and Zhang Y, are ranked among the top, reflecting the strong research contributions from China in this field. Among U.S. authors, Piette JD stands out with a high TGCS (181) and the highest h-index (67). Furthermore, although Bates DW has a relatively lower number of publications, he holds the highest h-index (132), further emphasizing his authoritative position in the academic community. Figure 5 illustrates the collaborative relationships between authors in the field. Through the author co-occurrence diagram generated by VOSviewer, several major collaborative groups formed among authors can be clearly observed. Specifically, the figure shows three main collaborative groups. Firstly, the red group centered on Rogers Anne, which involves multiple researchers and has a strong collaborative relationship. Secondly, the green group centered on O’Cathain Alicia, demonstrating a highly interactive collaborative network within. Finally, the blue group centered on Segar Julia, maintaining a relatively close collaboration, albeit on a slightly smaller scale. However, there is a lack of significant collaboration among the top 10 authors in terms of publications.

**Table 4:** Top 10 authors who published research papers.

Rank	Author	Publications, *n* (%)	TLCS^a^	TGCS^b^	h-Index
1	Deng N (China)	5 (1.47)	0	142	39
2	Lear SA (Canada)	5 (1.47)	4	53	34
3	Piette JD (United States)	5 (1.47)	2	181	67
4	Sakakibara BM (Canada)	5 (1.47)	4	46	20
5	Chen J (China)	4 (1.17)	2	83	15
6	Jayasena R (Australia)	4 (1.17)	0	73	11
7	Li J (China)	4 (1.17)	0	55	9
8	Varnfield M (Australia)	4 (1.17)	0	73	12
9	Zhang Y (China)	4 (1.17)	0	32	8
10	Bates DW (United States)	3 (0.88)	3	62	132

^aTLCS, total local citation score.^

^bTGCS, total global citation score.^

### Co-occurrence of keywords

**Figure 6:**
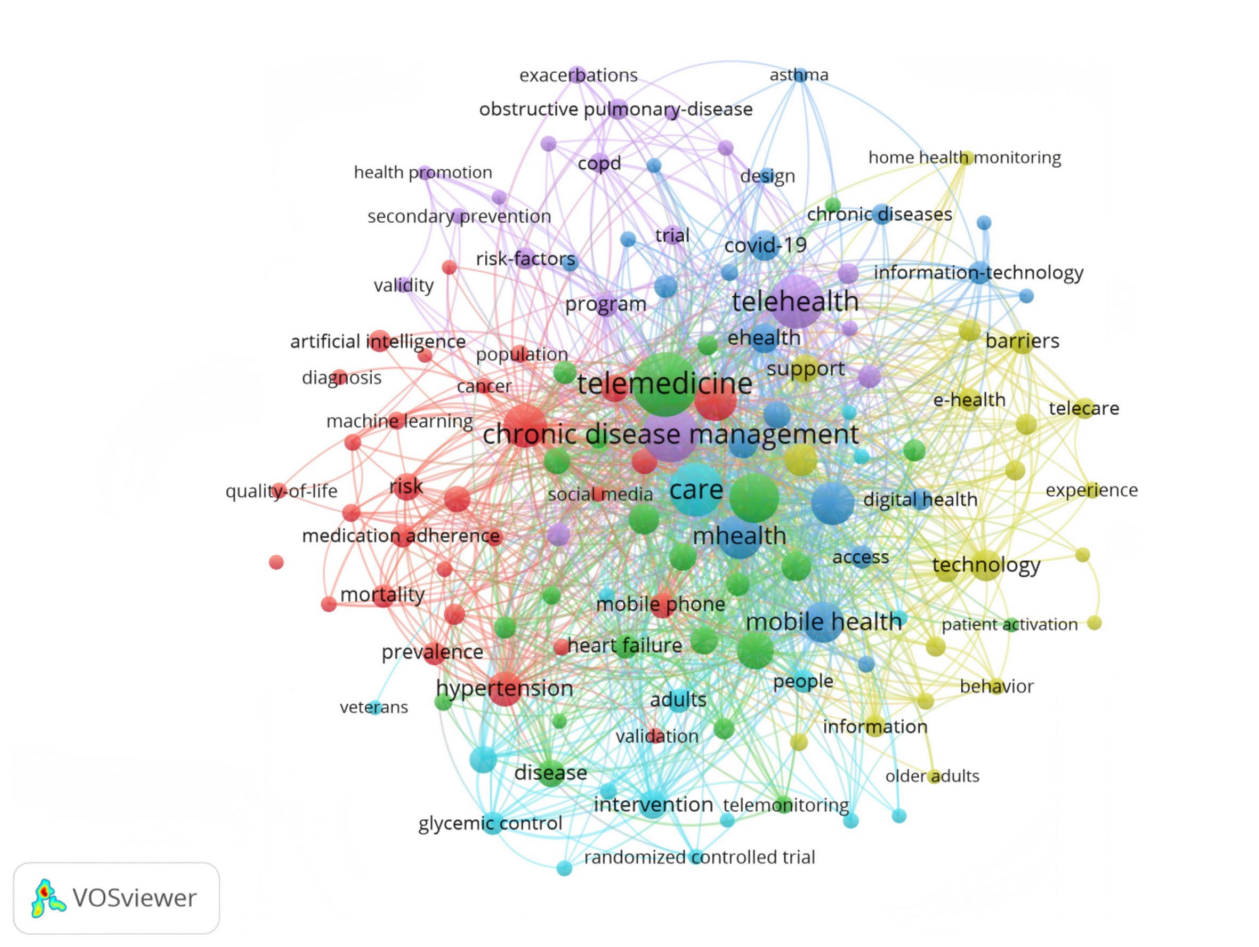
Keyword co-occurrence network.

Keyword co-occurrence analysis can identify potential research hotspots, providing insights into emerging trends and key focal points within a given field (29). In this study, the topics were identified through keyword co-occurrence analysis, with a focus on keywords that appeared more than five times. As shown in Figure 6, nodes in the graph represent different keywords, with their size corresponding to the frequency of occurrence. The connecting lines between nodes represent co-occurrence relationships, while line thickness reflects the strength of these relationships, indicating how often the terms appear together (30). Furthermore, the node colors (red, green, blue, and yellow) represent different clusters, each corresponding to distinct topics or subfields within the study. The graph reveals multiple dense clusters, indicating several major research directions and hotspots in chronic disease health management. The red cluster, with keywords such as ‘artificial intelligence’ ‘machine learning ‘diagnosis’ ‘prevalence’ and ‘mortality,’ primarily focused on the application of AI technology in chronic disease diagnosis, prevalence analysis, risk assessment, and prognosis prediction. The green cluster, represented by keywords such as ‘chronic disease management’ ‘care’ and ‘social media,’ reflected care strategies and the role of social media in patient support and management. The blue cluster, including keywords like ‘telemedicine’ ‘telehealth’ ‘mhealth’ and ‘mobile health’, underscored the importance of telemedicine and mobile health technologies in chronic disease management. Lastly, the yellow cluster, with keywords such as ‘technology’ ‘e-health’ and ‘digital health’, highlighted the role of digital and information technology in the development of chronic disease management strategies.

### Burst keyword detection

**Figure 7:**
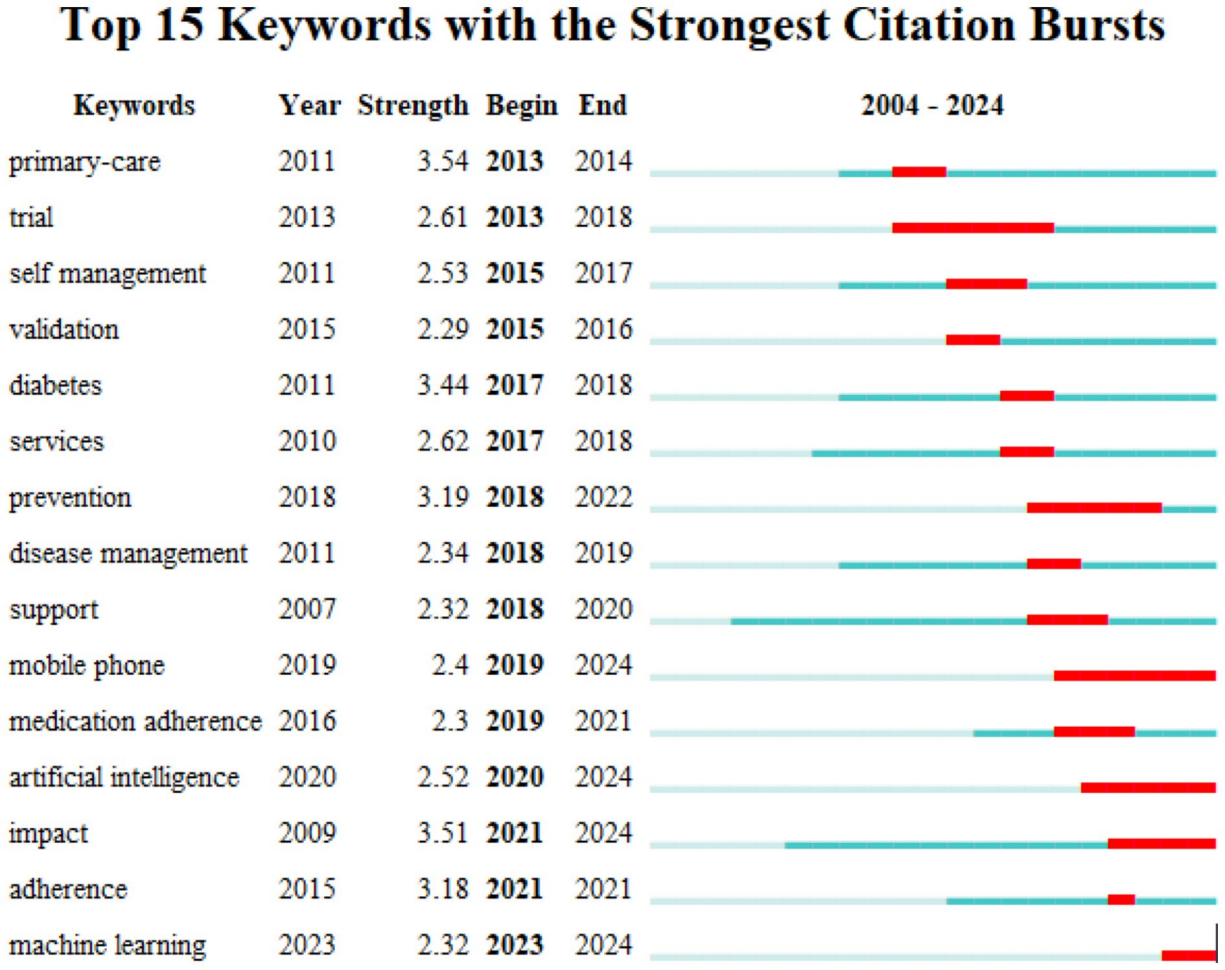
Top 15 keywords with the strongest citation bursts.

Keyword emergence is a technique used to track and measure changes in the frequency of specific keywords over time or under particular conditions. It plays a crucial role in identifying new trends, hot topics, or potential research directions (31). By analyzing keyword emergence mapping, researchers can highlight research hotspots and trends within a given field. This is illustrated by a significant increase in keyword frequency, represented by the red bars, across different years (32). As shown in Figure 7, the top 15 emergent keywords in the field are displayed, with “primary care” having the highest burst intensity (3.54), followed closely by “impact” (3.51) and “diabetes” (3.44). Furthermore, “trial” (5 years) and “prevention” (4 years) are notable as the keywords with the longest duration of burst activity. In addition, in the keyword mapping, “machine learning” has emerged as the most prominent keyword in recent years.

## Discussion

### Principal findings

Based on the bibliometric analysis results, this study systematically investigates the evolving trends in the application of AI for chronic disease health management over the past two decades. The findings reveal a notable and consistent growth in the volume of literature in this field. Specifically, from 2004 onwards, there has been a gradual yet steady rise in research focused on AI applications in chronic disease management. In particular, the period between 2013 and 2024 shows a marked acceleration in publication trends, indicating the heightened interest and investment of both researchers and clinical practitioners in this emerging area of study. Despite this positive momentum, it is crucial to recognize that the research on AI in chronic disease health management remains in its relatively nascent stages. While the rapid growth in publications is promising, the overall development of the field still lags behind in terms of quality and influence, as reflected in the declining TLCS in recent years. This suggests that although the quantity of research has increased significantly, the academic impact of these studies has not correspondingly improved, which could indicate challenges in the robustness, innovation, or clinical applicability of the recent studies. Furthermore, there is considerable room for further exploration and innovation in this field. The modest impact of early studies, coupled with the recent dip in citation influence, underscores the need for more high-quality research that can contribute meaningfully to both academic discourse and clinical practice. In this regard, future research should focus on addressing gaps in evidence, improving methodological rigor, and enhancing the translational potential of AI-driven approaches in chronic disease management. This will ensure that the expanding body of literature not only grows but also deepens the field’s impact on patient care and healthcare outcomes. In summary, while the increasing volume of research in AI and chronic disease health management is a promising indicator of progress, further efforts are needed to enhance the quality, relevance, and real-world impact of this research. These findings highlight the importance of continued scholarly and clinical attention to this burgeoning field, with an emphasis on fostering innovation that bridges the gap between AI advancements and improved patient care.

In addition to the above findings, this study further reveals that the United States has established itself as a global leader in research on AI technologies in chronic disease management. This dominance underscores not only the country’s significant research capacity and investment but also its extensive influence in shaping the direction of global research in this field. Nevertheless, it is equally important to acknowledge the substantial imbalance in the development of AI technologies for chronic disease management on a global scale. A detailed analysis of publication output and collaborative networks across countries and regions demonstrates that the United States accounts for over 50% of the global publications in this domain. In contrast, regions such as South America and Africa are markedly underrepresented, both in terms of research output and international collaboration. This discrepancy is further compounded by the fact that these regions engage in fewer scientific partnerships with developed countries, limiting their contributions to the broader discourse on AI in chronic disease management. This uneven development raises concerns about disparities in the application of AI technologies for chronic disease management across regions. Specifically, the lack of research and collaboration in underrepresented regions may hinder the global advancement of AI-driven solutions, ultimately affecting the equitable distribution of health management technologies. As a result, resource-limited countries and regions may struggle to access and implement AI-based innovations, exacerbating health inequalities. Therefore, to address these global disparities, it is essential that future research efforts prioritize and actively promote transnational cooperation, especially in resource-limited countries and regions. By fostering greater international collaboration, particularly with nations that are currently underrepresented in the research landscape, the global community can work toward more equitable development in AI-driven chronic disease management. This approach will not only enhance the global application of AI technologies but also contribute to more balanced and inclusive progress in global health management. In conclusion, while the United States and a few other countries continue to lead in AI research for chronic disease management, there is an urgent need to bridge the gap between developed and developing regions. Encouraging collaboration and providing resources for underrepresented regions are critical to ensuring that AI technologies benefit global health management equitably and sustainably.

By analyzing the co-occurrence of keywords, this study revealed that the main research focus of AI in chronic disease health management is centered around several prevalent chronic conditions, such as diabetes, hypertension, and chronic obstructive pulmonary disease (COPD). Specifically, within the field of diabetes, the application of AI predominantly focuses on early diagnosis, personalized treatment, and continuous monitoring. For instance, AI algorithms are capable of analyzing vast amounts of patient data, including blood glucose levels, insulin sensitivity, dietary habits, and exercise patterns, to predict glucose fluctuations and optimize medication dosages. Consequently, this approach not only enhances the accuracy of treatment but also effectively reduces the risk of complications (33). Additionally, AI-powered smart devices and mobile applications provide real-time glucose monitoring, along with personalized dietary and exercise advice, significantly improving patient self-management (34). Similarly, AI plays a pivotal role in the management of hypertension. By integrating patients’ blood pressure data, family history, and lifestyle habits, AI can predict the risk of hypertension and assist in the development of personalized antihypertensive treatment plans (35). Furthermore, integrating smart devices and remote monitoring technologies allows physicians to track blood pressure changes in real time and adjust treatment strategies accordingly, thereby improving patient compliance and treatment outcomes (36). Moreover, AI can analyze patients’ overall health data to identify potential risks of cardiovascular complications and provide early warnings, contributing to preventive care (37). In the context of COPD, AI demonstrates considerable potential in both diagnosis and disease progression monitoring. Specifically, by analyzing pulmonary function tests, imaging data, and symptom descriptions, AI algorithms can accurately diagnose COPD and determine disease severity (38). Additionally, AI assists in developing personalized treatment plans, which include optimizing medication usage, respiratory rehabilitation programs, and lifestyle modifications. Supported by smart devices, AI can remotely monitor respiratory status, detect early signs of deterioration, and intervene to prevent acute exacerbations (39). Although significant progress has been made in applying AI to manage conditions such as diabetes, hypertension, and COPD, it is important to note that AI applications in other chronic diseases, such as chronic kidney disease (CKD) and cardiovascular disease (CVD), remain relatively limited. Further research is necessary to deepen AI’s impact in these areas. Despite the growing body of research, systematic clinical validation and long-term follow-up data remain insufficient in chronic disease management (40). To fully realize AI’s potential, future efforts should focus on conducting more extensive clinical trials and accumulating long-term data to ensure the safety, efficacy, and sustainability of these technologies.

Another crucial application of AI in chronic disease management lies in risk management, specifically in predicting and preventing chronic disease complications. The literature emphasizes that risk management is an emerging key research focus in AI, as it substantially contributes to enhancing patient health outcomes through individualized risk assessments. AI-driven systems, in particular, can integrate various data sources, such as medical records, genetic information, lifestyle factors, and real-time monitoring data to conduct comprehensive risk assessments (41). Such multidimensional data analysis allows AI to predict the risk of chronic disease complications with greater accuracy, enabling healthcare providers and patients to take proactive preventive measures and effectively reduce risks. For instance, AI models have demonstrated significant benefits in predicting the risk of hypoglycemia in diabetes management. By analyzing a patient’s history of blood glucose fluctuations, medication use, dietary habits, and physical activity levels, AI systems can predict hypoglycemic risks in real time (42). When an elevated risk is detected, the system alerts both the patient and healthcare provider, suggesting necessary adjustments in medication or the need for dietary intervention, thereby preventing hypoglycemic events. This AI-based early warning system greatly enhances patient safety and improves quality of life. Similarly, AI models have shown promise in assessing the risk of acute exacerbations in COPD patients. By leveraging data such as respiratory rate, oxygen saturation, heart rate, and environmental factors like air quality, AI systems can predict the likelihood of exacerbations. Identifying high-risk patients early allows clinicians to adjust treatment plans or intensify monitoring, thereby reducing the risk of severe outcomes (43). Despite the promising potential of AI in risk management, several challenges persist (44). Foremost among these are concerns about data privacy and security, especially when integrating multiple data sources. Ensuring patient confidentiality is paramount. Additionally, the transparency and interpretability of AI models remain significant challenges. Healthcare providers and patients need to understand how AI-generated risk assessments are made to ensure that clinical decisions are well-informed. Moving forward, research should focus on improving the transparency and interpretability of AI models while simultaneously strengthening data privacy protection measures. Addressing these challenges is crucial for the safe and effective implementation of AI technologies in chronic disease risk management.

In recent years, the application of machine learning in chronic disease health management has gained increasing attention, as reflected by the rising prevalence of related keywords in bibliometric analyses. This research area has become a hotspot, with machine learning algorithms showing significant advantages, particularly in disease diagnosis, personalized treatment plan development, and patient behavior prediction. To begin with, disease diagnosis is a key application of machine learning technology. By analyzing large volumes of electronic health records (EHR), medical images, genetic data, and lifestyle information, machine learning models can significantly enhance the accuracy of early disease diagnosis (45). For example, in diabetes management, machine learning algorithms can integrate blood glucose records, genetic information, and body mass index (BMI) to predict the risk of developing diabetes, thus facilitating early intervention. Unlike traditional diagnostic methods, these models can automatically identify complex patterns, resulting in more precise diagnostic outcomes (46). Furthermore, machine learning holds great promise in developing personalized treatment plans. By leveraging individual patient data, these models can predict the effectiveness of various treatment options, aiding clinicians in tailoring treatment plans to the specific needs of their patients. For hypertension management, machine learning models can analyze drug response history, blood pressure trends, and lifestyle habits to optimize antihypertensive dosage and medication type, reducing side effects and enhancing treatment efficacy (47). In addition to diagnosis and treatment planning, machine learning has broader applications in chronic disease management, including resource optimization and health monitoring. Machine learning models enable healthcare providers to allocate resources more efficiently, predict which patients require more intensive medical interventions, and optimize healthcare resource utilization. Additionally, smart wearable devices combined with machine learning algorithms can continuously monitor chronic disease patients, detect abnormalities in real-time, and provide personalized health advice promptly (48). Despite the potential of machine learning in chronic disease management, the field is still in its early stages, and further exploration is necessary. In particular, challenges remain regarding model generalization and clinical validation (49). Current machine learning models may demonstrate unstable performance when applied to different populations and datasets, limiting their broader applicability in real-world clinical settings (50). Therefore, future research should prioritize developing more robust and interpretable machine learning models to enhance their adaptability across diverse scenarios. Additionally, large-scale clinical trials and data sharing will be crucial for validating the effectiveness of these models. Only through rigorous clinical validation can the safety and reliability of machine learning technologies be ensured, thus promoting their widespread application in chronic disease health management.

### Limitations

Since only the WoSCC database was searched, there may be some inherent bias in the results. Nevertheless, it is important to note that WoS is one of the most influential multidisciplinary academic literature indexing platforms worldwide, known for its authority and extensive coverage. Specifically, WoS includes over 12,400 high-impact academic journals from around the world, representing core literature across a wide range of disciplines. The high-quality journals and papers indexed by WoS offer reliable and comprehensive data support for this study. In addition, WoSCC provides a robust suite of citation analysis tools that facilitate in-depth exploration of academic impact and development trends within a specific research field. These tools offer valuable insights into current research hotspots, helping scholars better understand the evolution of their fields and providing useful references for future research endeavors.

## Conclusion

In conclusion, this study offers a systematic overview of AI research in chronic disease health management, analyzing current research trends and hotspots. Although publication volume has increased, the overall quality remains suboptimal, with significant limitations in study design, sample size, data quality, and methodology, which impact the reliability and generalizability of findings. We also found that much of the research is confined to specific countries or institutions, limiting broader technological advancements. To address this, we recommend stronger international and inter-institutional collaboration to exchange expertise, enhance research methodologies, and foster global innovation. Current AI applications mainly focus on managing chronic conditions like diabetes, hypertension, and COPD, using machine learning for risk prediction and management. Expanding research to other chronic diseases and deepening investigations in existing areas will be essential to improving AI’s effectiveness in health management. Despite challenges such as data quality, model accuracy, and clinical applicability, AI holds significant promise. Future research should optimize AI algorithms’ precision, adaptability, and interpretability, as well as improve integration with clinical decision-making. Addressing these challenges will enhance AI’s role in chronic disease management, improving patient outcomes and quality of life. In summary, AI has the potential to improve chronic disease management efficiency and effectiveness. Integrating these findings into clinical practice and ensuring high-quality research will promote wider AI adoption, benefiting patients and healthcare systems globally.

## Data Availability

The original contributions presented in the study are included in the article/supplementary material, further inquiries can be directed to the corresponding authors.
